# What are the optimal strategies to communicate the risk of poor air quality exposure to vulnerable groups?

**DOI:** 10.3389/fpubh.2026.1763393

**Published:** 2026-06-18

**Authors:** Clare L. Tolley, Audrey de Nazelle, S. Wilson, N. Hassan, R. D. Slight, S. P. Slight

**Affiliations:** 1 School of Pharmacy, Newcastle University, Newcastle Upon Tyne, United Kingdom; 2 Imperial College London, London, United Kingdom; 3 The Newcastle Upon Tyne Hospitals NHS Foundation Trust, Newcastle Upon Tyne, United Kingdom

**Keywords:** air pollution, health communication (MESH), public health, qualitative research, risk communication

## Abstract

**Background:**

Ambient air pollution is a critical global health concern. Individuals living with preexisting health conditions in urban and/or deprived areas and ethnic minorities groups are at particular risk. The WHO Global Air Quality Guidelines have highlighted the importance of effectively communicating the risk of poor air quality exposure to vulnerable groups so they can take actions to protect themselves. This study explored the optimal strategies to communicate this risk to vulnerable groups.

**Methods:**

In-depth, semi-structured interviews and focus groups with participants (*n* = 50) across a diverse range of vulnerable demographic groups living in North East England, to explore their perspectives around poor air quality and how to communicate this information.

**Findings:**

We developed a theory- and evidence-based framework for communicating the health impacts of poor air quality. We identified key components of a communication strategy focussing on the need for: tailored information on risks and harms, a range of inclusive and actionable recommendations, and considerations around the source (healthcare-provider, peers) and channel of communication (SMS, community-based).

**Interpretation:**

This study provides novel insights from vulnerable demographic groups supporting the need for air quality information to be accessible, understandable and tailored. Communicating via trusted sources, e.g., healthcare professionals and/or community-based peer networks is important. Further research should explore the impact of interventions on behaviour change, particularly across vulnerable groups.

## Background

Ambient air pollution is a critical global public health concern, estimated to cause 4.2 million premature deaths worldwide annually (1). A body of evidence has highlighted the impact of poor air quality on health across an individual’s life, contributing to respiratory and cardiovascular disease, diabetes, cognitive function, and reproductive outcomes (1–3). Individuals living with preexisting health conditions in urban and/or more deprived areas, and ethnic minorities groups, are at particular risk (4–7). Various legislation has been introduced to reduce air pollution (7–9). Key measures have included setting limits for major pollutants, monitoring requirements, and mandatory reporting. There is also an emphasis on using data to act such as imposing traffic restrictions, providing public health information and protecting vulnerable groups (8, 10). “The European Union’s Air Quality Directive” (Directive 2008/50/EC) requires member states to provide timely information to the public about actual or predicted exceedances to air pollution levels (8). The World Health Organisation (WHO) issued Global Air Quality Guidelines which highlighted the importance of effectively communicating the risk of poor air quality exposure to vulnerable groups so as to enable them to take actions to protect themselves (10). Risk communication, defined as the real-time exchange of information and advice from experts to people who face a hazard (e.g., members of the public at risk of exposure to air pollution), can enable people to make informed decisions to mitigate the effects of a hazard and take protective and preventive measures (11). The United Kingdom’s (UK’s) Department for Environment, Food and Rural Affairs (DEFRA); and UK Health Security Agency’s recent Air Quality Information System (AQIS) review, have further concluded a need for increased public awareness around air pollution effects and advice that is tailored and reaches vulnerable groups (12).

Previous studies have explored air quality communication interventions and identified a range of dissemination modes being used, as well as interventions focussed at the individual, organisational and to a lesser extent societal level (13, 14). Riley et al.’s literature review on air pollution communication, found information on poor air quality and the changes individuals can make to reduce their exposure can be shared in multiple ways such as smartphones, newspapers, radio, television, or public signage (14). Messages may include a range of recommended actions, such as changes to outdoor activity, driving-related behaviours (e.g., reduced idling in cars) or travel-behaviours (14). Cibin et al.’s recent review of 79 studies, however highlighted the need for further work to explore the effectiveness and role of different behaviours to prevent exposure and/or contribution to air pollution. The authors also suggested further work to investigate the effectiveness of interventions to promote civic engagement (e.g., advocacy and local projects) (13). Of note, this review only identified four studies that included health-vulnerable individuals and seven studies including individuals from lower socio-economic groups suggesting a lack of research that has focussed towards some of the most vulnerable groups. Indeed, recent studies have highlighted how current channels are “unlikely to reach the most vulnerable populations”. This is due to a combination of technical and sociodemographic influencing factors; for example, individuals who face digital exclusion may be unable to access relevant technology that may be used to provide information on poor air quality episodes. Furthermore, individuals with lower levels of literacy may be unable to understand and/or interpret this information, as it may contain technical terms (15). Research has also highlighted how information needs to be tailored for specific groups, ensuring that it is relatable, understandable and reaches those most at risk (14). Within this study we therefore aimed to explore *what* information vulnerable groups wanted to receive about air pollution, how and when they wanted to receive it, and the potential unintended consequences.

## Methods

### Study design

We conducted in-depth, semi-structured interviews and focus groups across a diverse range of vulnerable demographic groups to gain an understanding of individuals’ perspectives around poor air quality and how such information should be communicated.

### Setting and participants

Our study focused on the North East of England, where almost 20% of people are living in the most deprived areas of England and high rates of inequalities (16). Adults aged over 18 were eligible to participate; we purposefully included individuals from the following groups:

- Low to middle income groups or socioeconomic classes
- Different ethnic and racial groups
- Different religious beliefs
- Older adults (+65 years)
- People with low educational attainment
- People living with disabilities
- People who live in different geographic areas (rural/deprived areas/homeless individuals)
- People with multiple long-term conditions.
- People whose first language is not English.

### Ethics

This study was approved by the Research, Policy, Intelligence, and Ethics team at Newcastle University (reference: 2588/33982). Participants provided written and verbal consent and were offered a £20 voucher as a ‘thank you’ for their involvement.

### Recruitment

We used a purposeful snowball sampling approach, underpinned by extensive community engagement with voluntary organisations who operate in the North East region, local community centres and religious groups (17). Recruitment activities spanned over 18 months building connections and relationships with in excess of 15 different groups across different sectors (voluntary organisations and groups, religious communities, council and local authority groups/ initiatives, local community groups). We hosted project information sessions with these different groups and liaised with gatekeepers associated with some of these groups to understand individuals’ needs and preferences. This included understanding the best times to meet with potential participants (e.g., before or after a digital skills session), whether interpreters were required (i.e., the participant would prefer the interview to be conducted in their native language), or any individual requirements (e.g., arranging childcare to allow single mother(s) with young children to participate). A British Sign Language interpreter was in attendance if the potential participant(s) were known to live with hearing difficulties. Study researchers used these opportunities to provide information about their professional background and background to the study to build trust and engagement.

Alongside in-person information sessions, information about the project was circulated in paper and digital formats through relevant established national and local networks, e.g., Asthma Lung UK, VOICE Global, local community centre newsletters. Project posters were also displayed in public places such as community centres, places of worship and published in local newsletters. The posters were translated into different languages as appropriate (Supplementary Figures 1A,B). Information sheets (either digital or paper-based, Supplementary File 3) were also distributed in the project information sessions. Individuals who wished to participate got in contact either by phone or email using the information provided. Interested participants were asked to complete a short expression of interest form (either digitally or written, with support from a researcher/ interpreter, at the participant’s request), this collected demographic information, to help identify eligibility and monitor diversity (40).

### Data collection

The information sheet explained the different data collection methods and what they involved. Participants chose to participate in either a semi-structured interview or focus group, facilitated by a study researcher with post-graduate qualifications [CT, NH or SW (female)] between Oct 23 – Feb 24. Data collection was conducted in the participant’s preferred language [two interviews were conducted in Arabic with a native Arabic speaker (NH), or with a sign language interpreter with speech to text operator]. A flexible interview or focus group topic guide (Supplementary File 2) was used alongside a stimulus power point presentation, which was informed by the Capability Opportunity Motivation-Behaviour (COM-B) model to ensure exploration of key focus areas of interest, e.g., participants knowledge of air pollution and skills/ ability to adapt their behaviour (capability), whether participants were able to make changes to reduce their exposure and what factors influenced this (opportunity) and to understand key motivating factors such as perceived health susceptibility, and trust in the information provided (motivation) (18). The stimulus included imagery of strategies to communicate air quality information and risk mitigation strategies, and interview question prompts. Questions sought to establish baseline understanding of poor air quality, what information individuals want to receive about elevated levels, when, where and how they would like to receive it, and potential benefits and barriers of each. We also explored the content of the messaging. Interviews lasted between 30–60 min, and focus groups between 1–2 h.

Fifty participants took part in either an interview (*n* = 6) or focus group (*n* = 8, which included between 2 to 10 participants); these were audio recorded (with consent) via a video conference software (Microsoft Teams or Zoom), or digital recorder if conducted in person. In person data collection took place within various local community centres and local authority. The recordings were transcribed verbatim, using the transcribing function, or by a third-party transcription service. All transcripts were cross checked for accuracy and anonymised; female (*n* = 33, 66%) and male (*n* = 17, 34%). Participants were from a range of different ethnic backgrounds, including white (*n* = 27, 54%), Asian (*n* = 11, 22%), Black African/ Caribbean/ Black British (*n* = 4, 8%), Black-Portuguese (*n* = 1, 2%), mixed ethnicity (*n* = 1, 2%) and British Bangali (*n* = 1, 2%), and spoke one of seven different preferred languages. It was the first time most individuals (*n* = 30, 60%) participated in research. Participants were either Christian (*n* = 13, 26%) or Muslim (*n* = 13, 26%). Most were educated to A-level standard (equivalent to US Advanced Placement, European Baccalaureate) or above, and seven (14%) had no qualifications at all. Almost a third of participants (*n* = 16) had a chronic health condition.

### Analysis

Qualitative data collection and analysis were iterative, allowing themes to be generated, interpreted, explored, and disconfirming evidence identified. The use of interviews and focus groups facilitated triangulation to identify convergence within the data. We used a two stage approach to analysis. Firstly, the data were analysed following Braun and Clark’s six phase approach for reflexive thematic analysis (19). We took an initial inductive approach to coding by one researcher (CT) and theme development, underpinned by a constructivist epistemology (i.e., knowledge is actively constructed through social interactions, experiences, and interpretation) to ensure the themes were meaningful and relevant to the research questions (19). Themes were discussed among team members and continually refined and applied systematically across the data using the computerized software N-Vivo (V14 QSR International). A summary of the inductively derived themes were then compared and contrasted with a previously developed framework developed by our team (Riley et al.,) as part of a prior review (14) (deductive analysis). This literature-based framework outlined key domains of recommendations/ target areas relating to air pollution communication approaches relating to the: message channel, receiver and source that may support behaviour change (14). This framework was chosen due to clear synergies in both study’s aims and findings (14). To provide a theory and evidence-based grounding to the analysis, we evaluated how the emerging themes could be structured along the dimensions identified in Riley et al’s literature-based air pollution communication framework, which is also informed by the COM-B model. Following this structure enabled a systematic approach to compare current findings with the existing body of evidence on air pollution communication, including highlighting contributions to what had been deemed by Riley et al to be promising but under-studied avenues for engagement. Rigour was upheld during all stages of this study by completing the Consolidated criteria for reporting qualitative research (COREQ) checklist (Supplementary File 1). The research team were experienced qualitative researchers at various career stages, with academic backgrounds in public health and pharmacy. This multidisciplinary approach mitigated bias from each researcher’s own interests and assumptions (based on their different epistemological and ontological stance) and ensured a comprehensive understanding of participants’ experiences.

Our separate Patient and Public Involvement group (six persons) were involved in all stages of the study and protocol development. The group included individuals from different backgrounds with representation of diverse ethnicities, nationalities, living with health conditions, employment and caring responsibilities. The group were vital to ensure that individuals with lived experience could contribute to the study design and support production of meaningful results. The group reviewed study documents and made suggestions on how to strengthen our recruitment approach. We also discussed our results with the Patient and Public Involvement group to provide a ‘sense check’ and ensure our interpretations accurately reflect the views of vulnerable populations.

### Findings

**Figure 1:**
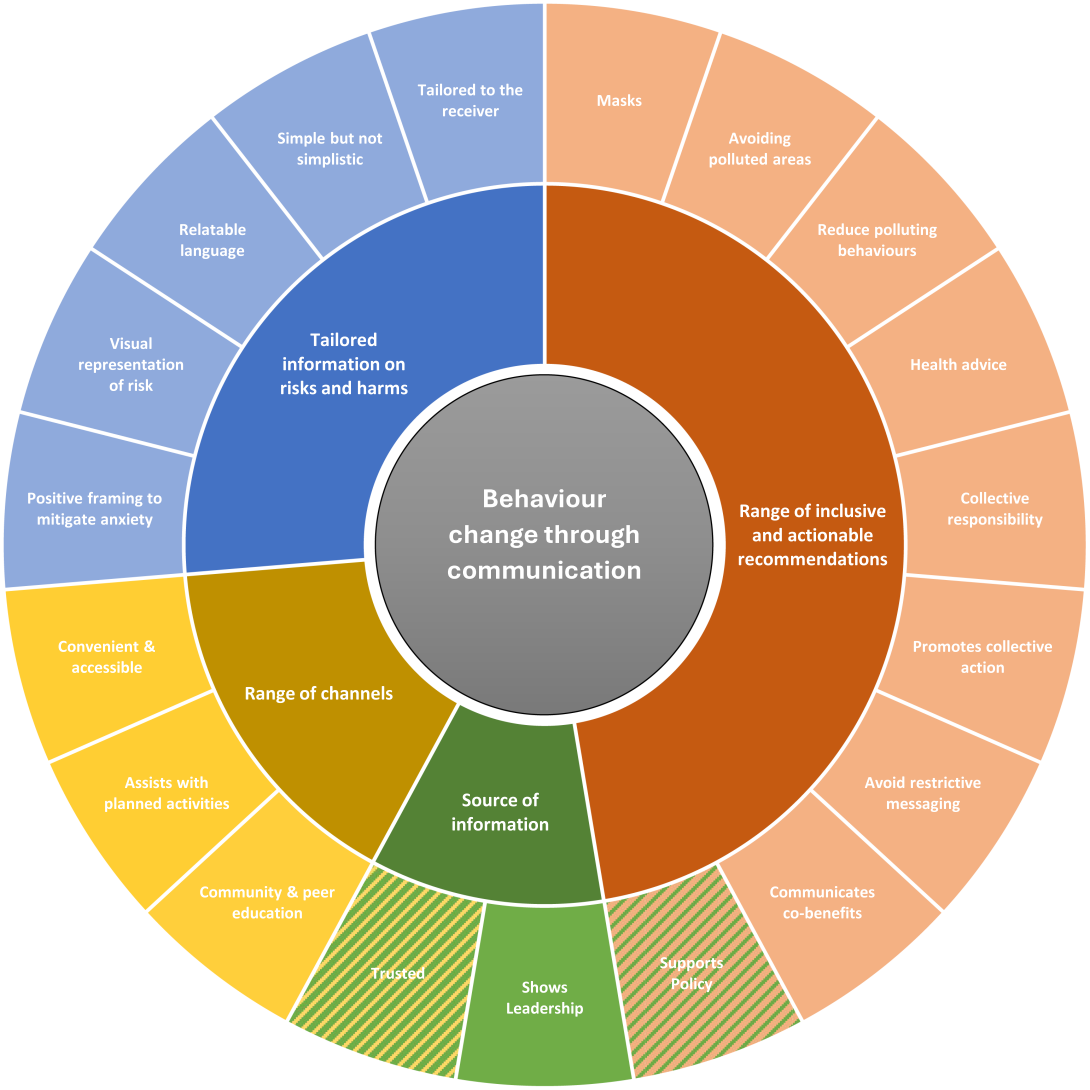
Components of a communication strategy to target behaviours to reduce AQ exposure and/or mitigate the effects of exposure.

Four main components (and 19 sub-themes) of a communication strategy were identified (Figure 1), and are discussed in detail.

### Comparison with Riley et al.’s literature review derived framework

Our communication strategy build on Riley et al.’s (14) recommendations for air pollution communication, refined using findings from the current analysis. The key components of this framework are the Message, Source, Channel, and Receiver. The outer ring recommended collaborating stakeholders (e.g., policymakers, businesses, community groups, researchers, etc.) Our findings resonated well with most of the 17 recommendations identified the Riley et al.’s framework. Our respondents however did not highlight the need to activate social norms and identities; to use emotions, personification, metaphors, and human centred-communication to engage the public. On the other hand, our respondents contributed further insights into how risk should be presented using visual approaches and emphasised the importance of dissemination information to vulnerable groups, such as through community and peer-based groups. We simplified Riley’s framework to highlight more prominently 4 key components of a communication strategy, shown in the centre of Figure 1 and detailed in the following four sections. We expanded Riley’s 17 recommendations to the 19 specific solutions identified by our respondents shown in the outer circle of Figure 1. This included unique aspects that are specific to the channel used, highlighting a role for a communication tool to assist with planned activities and importance of community and peer education particularly when engaging with vulnerable groups. Our revised framework also provides additional detail on the range of inclusive and actionable recommendations, which have been considered by diverse groups, including some of the most vulnerable in society. This expands current awareness of the acceptability and feasibility of different approaches to reduce exposure and/or contribution to air pollution.

#### Tailored information on risks and harms

Some participants understood concepts such as particulate matter (PM) and health impacts, while others incorrectly referred to air pollution as an air borne germ that children could bring home from school. One participant described seeing heavy traffic in a nearby road, but did not usually associate this with elevated air pollution: “even though I can see the A1 (major UK motorway) from my house. I can see how busy it is, it doesn’t stick in my mind like, *“Oh, that’s a lot of air pollution going on there” (P27, Focus Group 5)*. Individuals from all backgrounds felt it was important to understand these risks and harms, particularly in terms of the impact on their health and the environment.

Many participants felt it was important to find the right balance between making the information understandable and sufficiently simple (*but not too simplistic*). Participants described how health risks and harms should be translated into tangible outcomes such as *“at this level, then people with weak chests might have difficulty breathing” (P42, Focus Group 8)*. Participants wanted tailored messaging to inform actions based on their own individual health risk: *“What’s a dangerous level for one person, isn’t a dangerous level for another” (P43, Focus Group 8).* Participants proposed representing risk visually, using a traffic-light signal, and providing details on whether the levels had increased or decreased over time. Caution was advised against too much technical information because *“once you start going into 97 particles per million whatever’s (…) nobody will have the squidgy what’s going on” [P48 (Focus group 8)]*. Participants recognised the complexity of air pollution messaging and also raised concerns around provoking fear:

*“People might start to panic (…)* there is the risk that *“people will have to worry about their health instead of [focussing on how] to take prevention” (P15 Focus Group 3).*

Participants stressed the importance of positive message framing, and balancing the risks and benefits of taking a particular action, e.g., staying indoors.

#### Range of inclusive and actionable recommendations

Participants were unsure about how to reduce their exposure and/or contribution to poor air quality. Some participants, who had lower educational attainment levels or were from minority ethnic groups, discussed how *“we don’t know what to do” (P12, Interview)* if levels elevate. One participant, who was an asylum seeker, suggested *“buy[ing] some drugs from the pharmacy and just keeping [them] in the house” (P15, Focus Group 3).* Other participants, who were educated to a higher level and/or affluent, proposed buying and/or using air filters in their home, or keeping medicines such as inhalers on them when they left the house. Participants also suggested providing information about *how* to avoid polluted areas with affordable alternatives. Many participants appeared willing to adjust their travel plans, with one suggesting that they would check *“pollution levels on the go” (P8, Focus Group 2)* to avoid more polluted areas. Another participant avoided certain polluted areas because his daughter had asthma, while a third participant described how she had *“a child with a heart problem so I have to make sure that I am protecting her” (P30, Focus Group 6).* One participant described living near the airport and being advised to move house by a healthcare professional, their doctor, as the poor air quality *“was really affecting his [participant’s brother’s] development” (P25, Focus Group 5).* Some individuals also preferred to take a quieter or less congested travel route with the added benefit of increasing exercise levels, others were happy to take “a slight detour” if it did not delay them too much.

Most participants from all different backgrounds wanted to receive an actionable recommendation, such as *“whether to wear a mask or not” (P7, Focus Group 1).* Some individuals routinely wore a mask or face covering to reduce their exposure to unpleasant smells, with one participant explaining how *“if I am not wearing a mask, I just have to use whatever I have got to cover myself (…) a scarf or whatever” (P29, Focus Group 6).* Others were less amenable to wearing a mask, especially those with respiratory conditions who *“struggled to breathe anyway, never mind trying to breathe through a mask” (P3, Focus Group 1).* Wearing a mask could also be problematic amongst individuals who were deaf/hard of hearing because *“they rely[relied] on lip-reading for communication” (P47, Focus Group 8).* One participant reflected on how they *“wouldn’t choose to wear a mask, but if it protects me (…) I would promote it to my family” (P29, Focus Group 6)*.

Some participants highlighted the importance of collective responsibility and/or collective action to reduce air pollution exposure. For example, participants wanted to know the source of pollutants (e.g., local factory) and whether levels were rising. Participants admitted that they needed prompting with *“ideas and active things that we can do” (P31, Focus Group 6)* to reduce polluting behaviours. Some suggested collective initiatives such as the *“road is closed and only cyclists and pedestrians are allowed” (P21, Interview)* and encouraging school children to travel via *“a walking bus” (P21, Interview).* One participant was sceptical about changing their individual behaviour, without this collective action:

*“if I start cycling or walking to work, I’m just one person. So, what about the other 10,000 people that are all driving their cars?” (P23, Focus Group 5)*

Participants occasionally expressed frustration towards local and national governments and provided suggestions about what they thought local governing organisations could do, including implementing driving restrictions, increasing green spaces, and better charging infrastructure for electric vehicles. Participants felt it was important that organisations and policymakers communicate the actions that they are taking, with one suggesting that factories should *“put money into greenbelt areas or (…) try and rewild areas” (P10, Focus Group 2).* Other participants suggested raising question(s) with their local councillors to understand the reasons for poor air quality and lobby for improvements.

#### Source of information

Participants stressed the importance of delivering air quality information and actionable recommendations through trusted routes, such as the local authority, NHS and/or Public Health. Educating young people within schools was considered important, with one participant explaining how the activities that their three children learned *“sticked with them and still love it and comply with it” (P21, Interview).* Another participant described how it would be useful for governing organisations to provide information that can *“tell us what is the permitted level for this nitric oxide’ (…) and if it is increased it can cause a…b…and c….” (P22, Interview)* Participants felt that health and social care authorities *“could do a lot more in relation to encouraging people about air pollution and acknowledge air pollution[risks]” (P1, Interview).* Participants suggested that *“most people […] would trust” (P1, Interview)* information from healthcare affiliated sources, e.g., public health bodies or individual practitioners.

#### Channel of communication

Participants described how the timeliness of the message should influence the chosen channel. For example, if a change in air pollution levels was expected imminently, text message-based alerts or notifications were viewed as acceptable. This approach was preferred in those from different age groups, ethnic backgrounds, socioeconomic statuses, and those with different health conditions, with mobiles perceived as convenient and accessible*: “we hold our mobiles in [our] hand all the time and if we need anything we check our mobiles” (P22, Interview).* However, if the need to communicate was less urgent, participants discussed more unobtrusive approaches, such as checking a website. Applications (or ‘Apps’) were noted to offer additional usability features, such as allowing users to “*opt out [of the notifications] if you want[ed] to” (P6, Focus Group 1)* or change the font to a different colour or size. It was felt that push notifications could catch people’s attention and encourage them to use it more. One participant explained how they had a weather app on their phone and “*check[ed] it every day to see if it’s raining or whatever” (P50, Focus Group 8);* they liked the passive nature of this. However, others were reluctant to download additional apps due to a lack of storage space on their device.

Community-based communication and peer education was suggested by individuals from ethnic minority groups and those with limiting conditions, where there was a strong sense of responsibility around caring for others. One participant, who did not own a phone, explained how she got *“information from [her] family” (P30, Focus Group 6).* Television was perceived as useful alongside social media updates, such as Facebook. One older participant however questioned the content *on social media* because *“you don’t know what’s true and what’s not”,* and would *“rather have a text” (P13, Interview)* from a trusted source. Some participants suggested advertising *“in the waiting area in hospitals” (P22, Interview)* or on *“big screens in the streets” (P13).* Another participant recalled how in another country, *“there was a video screen [showing health promotion] on all the buses” (P46, Focus Group 8)* and questioned *“why we don’t have something similar here?” (P46, Focus Group 8).* A participant suggested placing air pollution monitors in different locations within a local area and sharing the recordings in real time with residents, to help increase awareness and collective action: *“this recording [could be seen] online and even on television to make people realise just what’s going on” (P1, Interview).*

## Discussion

This research provides a theory- and evidence-based framework for communicating the health impacts of poor air quality, particularly to vulnerable population groups (14, 18). Our findings have highlighted how participants wanted to learn more about poor air quality, the related risks and harms for their health, and changes that can be made at an individual and societal level. Air quality information needed to be readily available, easily understandable and delivered in different ways for vulnerable groups. Air quality information should be disseminated by trusted sources, e.g., healthcare professionals. Social networks could promote public awareness and encourage behaviour change. Individual action could also be framed within a broader context of community change.

### Education and communication on risks and harms

Study participants felt uninformed about the health effects of poor air quality. This was particularly evident in individuals from ethnic minority groups, individuals of lower socioeconomic status and/or lower educational attainment. Research suggests over half of Europeans felt uninformed about air quality in their country (20), and a recent UK DEFRA report highlighted individuals were unsure how to avoid harmful levels of air pollution (12). We found individuals wanted information to be understandable and personalised. A recent review of the UK’s Daily Air Quality Index (DAQI), which groups data into four categories (low, moderate, high and very high), found that the information was technically accessible and understandable. However, the information was not personalised and was only directly accessed by a small proportion of UK residents (21). The UK’s AQIS report called for the risks of poor air quality to be more effectively communicated to vulnerable individuals and their carers through suitable channels, including trusted messengers, like healthcare professionals, relevant health and care services or TV/weather broadcasts, or indeed community-based peers/ contacts (12). This could involve health care professionals engaging in meaningful discussions with vulnerable individuals around the health impacts of poor air quality and what actions they can take. Public Health Research has found that peer supporters, with the right knowledge and skills, can build connections and promote discussions around health and wellbeing in the community. This approach may also reduce health inequalities by removing hierarchical barriers (e.g., during service-user/healthcare professional consultations where individuals do not feel comfortable discussing barriers to change) (22). Communicating information on the risks and harms of air pollution requires careful consideration of how to avoid unnecessary distress, which could increase information avoidance, or ‘alert fatigue’ whereby receiving high volumes of alerts or advice can actually decrease recall of message content (23, 24). Furthermore, messages should not place responsibility on vulnerable individuals who may lack the means to take protective measures. Communication interventions, therefore, should build trust by signposting to functioning and accessible services, and acknowledge uncertainty. Community engagement is also key, to ensure interventions are appropriate and feel “community-owned” (25).

### Tailored actionable recommendations

Our findings showed that protective actions need to be acceptable, timely, feasible and affordable and include strategies at the individual and societal level. Participants in our study described a wiliness to wear masks, if needed. Although wider evidence has shown some benefits of wearing masks, the quality and how it has been fitted were also important considerations that must be communicated appropriately (26).

This study found that participants appeared willing to take an alternative travel route (quieter) or mode of transport (cycling), to reduce their exposure to air pollution, if it benefited them. Thus, future air pollution communication systems should communicate the co-benefits of actions that people value. To support these changes, our work and others, calls for public health officials and urban planners to promote active travel routes that are both safe and acceptable (12, 14, 27). Proposed interventions that have overlapping benefits across sectors (e.g., health and environmental policy) should also be considered (14, 28, 29). Our findings repeatedly call for collective action, with participants interested in understanding the specific sources of air pollution, and how key polluters might offset their emissions.

### Channel

Our findings support using a range of communication approaches to disseminate air quality information and promote equity. SMS based health messaging was viewed by participants as a simple and accessible approach that could reach people across different socioeconomic groups and, unlike other prompts, were less likely to be ignored. SMS messaging has been used as part of public health initiatives, including physical activity promotion and chronic disease self-management, resulting in improved clinical outcomes (30). Future research should explore messaging via this channel and whether information is acted upon over time (31). Furthermore, the risks of digital exclusion must be adequately considered and mitigated in future digitally mediated approaches (32). Some participants in our study showed interest in receiving real-time alerts, including information from local air quality sensors. Transport or journey planning apps are routinely used by the public and could potentially be expanded to provide information and build awareness of health impacts (33).

## Strengths and limitations

This study included a diverse participant sample from North East England and data collected until thematic saturation was reached (i.e., when no new insights or themes emerged form data collection). This was assessed by reflecting on the quality of the interviews and focus groups (e.g., duration and depth of discussion) and the occurrence of themes and concepts in the data during team meetings (34). However, these findings may not be generalisable to other regions or groups of individuals. There is also a need to consider the transferability of our findings to other contexts. For instance, trusted sources identified in this study included healthcare providers, however this may vary between settings. Similarly, the extent to which organisations and policymakers can communicate the public health information or actions they are taking to reduce or mitigate the risks of air pollution, is likely to vary. This depends on organisational and system-level differences in culture, politics and resources. Other findings such as those focussed on providing convenient and accessible information, and providing tailored information on risks and harms, meanwhile are likely more ubiquitous across contexts.

Data was collected via semi-structured interviews and focus groups, including translators and British sign language interpreters, where relevant. Throughout data collection and analysis, notes were recorded of reflections to support analysis and interpretation (35). Data collection was conducted during a period of industrial strike action across local public transport, potentially influencing discussions. While this study focussed on the views of the public, future work could explore approaches taken by organisations or policy makers to mitigate the health impacts of air pollution and the barriers and facilitators to these at the societal level. We acknowledge that most participants were educated to A-level education or above; educational attainment likely influenced risk perception and the subsequent actions. Thus, further work could focus on air pollution risk communication particularly in those from lower levels of educational attainment or literacy (36). Our approach did not utilise a communication model, however we did explore key components of communication models, e.g., the sender, message, channel, the recipient and factors influencing their behaviours (37). Further work might apply relevant theory around communication, particularly to analyse future communication prototypes (38). Relevant theory, e.g., Theory of Planned Behaviour, may further support the development of an effective communication strategy, in future work (39).

## Conclusion

This in-depth study provides a theory- and evidence-based framework for communicating the health impacts of poor air quality, particularly to vulnerable population groups. Air quality information needs to be accessible, understandable and tailored, and should be communicated via trusted sources, e.g., healthcare professionals and/or peer networks. Future research should explore the role of SMS-based approaches, alongside strategies to promote education and awareness about the impact of poor air quality. The impact of interventions on behaviour change, particularly in vulnerable groups must also be explored.

## Data Availability

The original contributions presented in the study are included in the article/Supplementary material, further inquiries can be directed to the corresponding author/s.
