# Supplementary material for: What are the optimal strategies to communicate the risk of poor air quality exposure to vulnerable groups?
Source: Front Public Health. 2026 Jun 18;14:1763393. doi: 10.3389/fpubh.2026.1763393 (PMC13326810; doi:10.3389/fpubh.2026.1763393)
Supplement: Supplementary file 2 [file Supplementary_file_2.docx]

**Interview guide**

I would like to start by thanking you for making time today to take part in this interview. I am interested in hearing your thoughts on air pollution and how we might be better able to communicate information about air pollution. Do you have any questions below we begin?

Would it be okay if I recorded our conversation? Everything you say will be kept confidential.

1. What comes to mind when you think of the term “air pollution”?
2. What impacts do you think air pollution has on health?
3. Have you ever changed what you were doing because of concerns or worries about air pollution?
   - - E.g., Changed your route to work / avoided a polluted area?
     - What influenced you to make that change?
       - Probe into factors influencing capability, opportunity and motivations to make change
     - How did you know if the air quality is good or bad?
4. We’re now going to ask a few questions about giving information about air pollution, and what you would find helpful.
5. Would it be useful for you to know if the air quality is good or bad?
   1. How do you think you might use this information?
6. **What** sort of information would you like to have?

Note for interviewer: give prompts if the participant struggles to come up with suggestions.

- 1. e.g., when will the level rise? By how much? Why is this information important for you?
  2. **location** of high or lower levels of air pollution?
  3. **timing** of high levels of air pollution?
  4. **impact of air pollution** to your health or others in the neighbourhood?
  5. **streets or travel routes** with lower air pollution levels?
  6. What can I do to protect myself from air pollution?
  7. What can I do to reduce my exposure to air pollution?
  8. What can I do to reduce air pollution?
  9. Knowing what **support or services are in place?**

1. **How** would you like to receive information about air quality/ air pollution levels?
2. What are your thoughts about these different approaches? (Present images of communication approaches to encourage thoughts if the participant doesn’t come up with any)

- **When** would you like to receive this information (week, day before, day of…?)
  1. Why is this way of giving information better or particularly helpful for you?

1. Once received (air pollution level will be high), would it influence your decisions / actions you might now take?
   1. e.g., stay inside, etc.
   2. What might prevent you from taking this action?
2. And finally, does it matter who provides this information, for example whether it is the local council, the NHS, researchers from universities…n?
3. Why?
4. Any other reflections on what we have discussed today?
