# Supplementary material for: What are the optimal strategies to communicate the risk of poor air quality exposure to vulnerable groups?
Source: Front Public Health. 2026 Jun 18;14:1763393. doi: 10.3389/fpubh.2026.1763393 (PMC13326810; doi:10.3389/fpubh.2026.1763393)
Supplement: Supplementary file 3 [file Supplementary_file_3.docx]

**
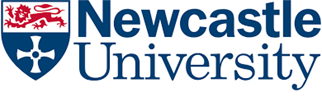
Participant Information Sheet**

**The HEALTH Project**

**Project background and aim:**

Air pollution has a serious impact on our health. Research has found that up to 36,000 deaths occur each year in the UK because of air pollution. Poor air quality can affect the whole body, including the lungs, eyes, nose, throat, and heart. The most vulnerable people in our society, like those with existing health conditions and from low-income communities, are affected more often. Information about air pollution levels, health risks, and steps a person can take to improve their health must be communicated to people in the right way, at the right time, and with the right content. Electronic devices can help. However, some people may not have access or the skills, to use and benefit from these devices. Research is needed to work with a range of different people to develop messages about air pollution levels.

In this study, we would like to understand how best to communicate important information about high air pollution levels to support individuals to improve their own health and wellbeing.

**Is this project right for you?**

Anyone over the age of 18 is welcome to take part. We are especially keen to involve people from:

- low to middle **income** groups or socioeconomic classes
- different **ethnic and racial** groups
- different religious beliefs
- older adults (+65 years)
- people with **low educational attainment**
- people living with **disabilities**
- people who live in different **geographic areas** (rural/deprived areas/homeless individuals)
- people with **multiple long-term conditions**.
- people whose **first language is not English**.

If you would like to have a chat about the project, please contact one of our research team (see contact details at the end of this document). We will arrange for an interpreter or written translations in other languages or formats if needed.

**What does the activity involve?**

If you agree to take part, a member of our research team will contact you. They will ask you to fill out a short (5 minute) questionnaire and give you the option of taking part in a one-to-one chat (interview) or as part of a group discussion (focus group). Please see the picture below for more information about the pros and cons of taking part in an interview and focus group. The research team are happy to discuss these options with you in more detail.


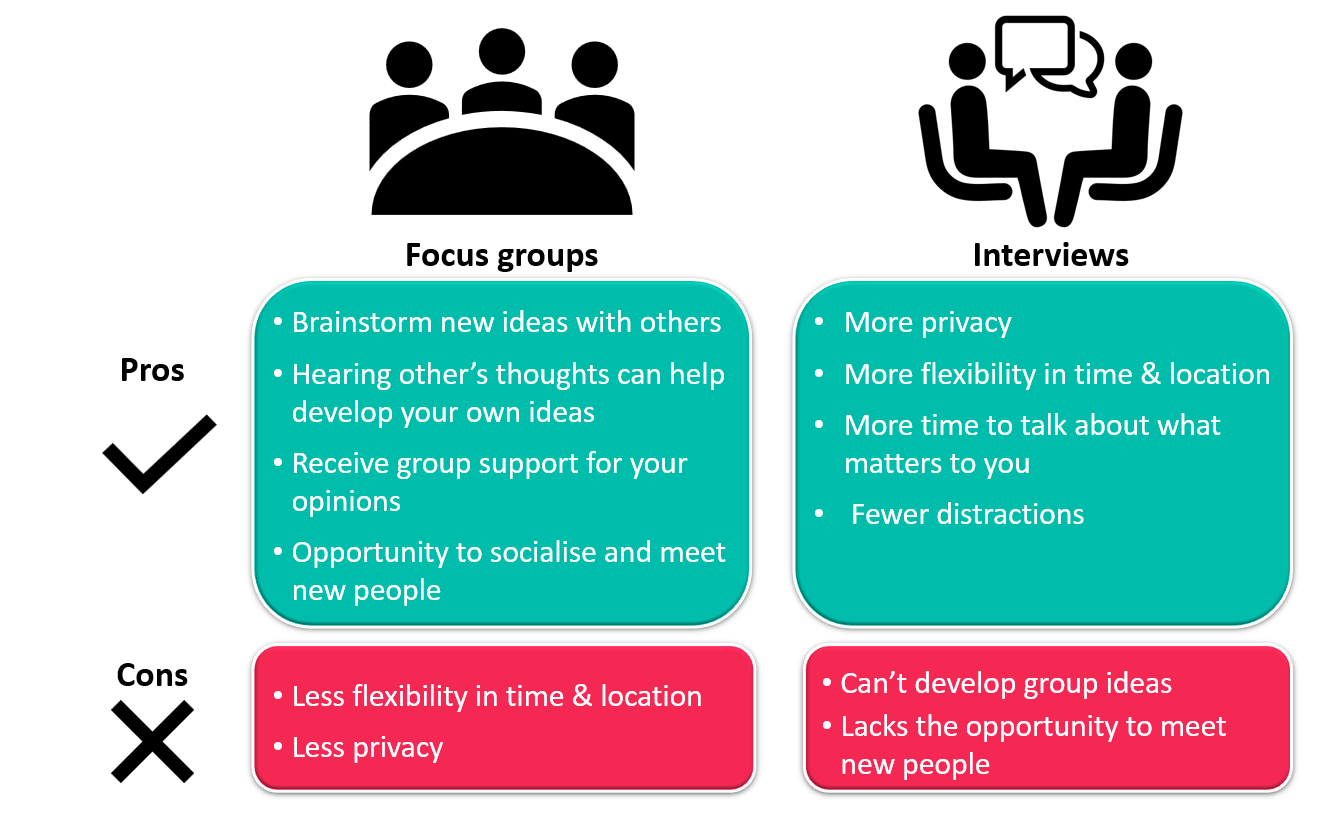


We would like to explore the topic of air pollution, its impact on health, and on how we communicate information about it.

The interview will take approximately 40-60 minutes and if you would prefer to take part in a focus group it will last approximately 60-90 minutes. A member of our team will contact you to arrange a suitable time and place.

If you express an interest to take part in this project but we are unfortunately unable to include you at this time, we will invite you to join a community network group where we will share updates on the research and post future opportunities to get involved.

**What are the benefits of taking part in this activity?**

By taking part in this activity, you will help the HEALTH project team understand your thoughts on air pollution, and how we might be better able to communicate information about it. You will also receive a £20 voucher for taking part (or we can make a donation to a charity/ local community group if you prefer), reimbursement for any travel costs, and a summary of the key findings from this project.

**What are the potential disadvantages of taking part in this activity?**

We do not anticipate any risks to you because of taking part in this project. Participation will require some of your time and as part of the questionnaire, focus group and/or interview will discuss your thoughts and experiences about air pollution and health, which some may find upsetting. If you are distressed, we will suspend the questionnaire/ interview and will take steps to signpost you to further relevant support, as needed.

**Will my taking part in this activity be kept confidential?**

If you decide to take part, data that you have provided through the survey and interview or focus group will be kept confidential. The interview recordings will be transcribed, using the transcribing function on Teams or Zoom and cross checked by a member of the research team, or using an approved third-party transcription service (e.g., UK Transcription https://www.uktranscription.com/) which will also be checked by a member of the research team. You will not automatically be expected to take part in any future engagement activities. You will only be identified to the researchers by a unique study number, and all of your data will be kept securely. You will not be identified in any reports, publications or presentations that include your data.

**What will happen to the results of the engagement activity?**

We will use the information collected from the interview or focus group to help design an appropriate communication strategy to provide people with personalised information to reduce their air pollution exposure. Outcomes will also be shared at conferences and in publications. You are free to withdraw from this study at any time without giving any reason. However, if you decide to withdraw, any data that has been provided up to that point will be included as it may have been incorporated into the analysis.

**Who is the sponsor and data controller for this research?**

The sponsor is the individual, company, institution or organisation that takes on legal responsibility for the initiation, management and/or financing of the research. The sponsor for this research is Newcastle University

Newcastle University will be using information from you in order to undertake this research study and will act as the data controller for this study.  This means that Newcastle University is responsible for looking after your information and using it properly.  When we use personally-identifiable information from people who have agreed to take part in research, we ensure that it is in the public interest.  Your rights to access, change or move your information are limited, as Newcastle University needs to manage your information in specific ways in order for the research to be reliable and accurate.  If you withdraw from the study, Newcastle University will keep the information about you that has already been obtained.  To safeguard your rights, the minimum personally-identifiable information will be used.  You can find out more about how Newcastle University uses your information at https://www.ncl.ac.uk/data.protection/dataprotectionpolicy/privacynotice/ and/or by contacting Newcastle University’s Data Protection Officer (Maureen Wilkinson, rec-man@ncl.ac.uk).

We will use your name and contact details [telephone number, email address] to contact you about the research study.  We will use your preferred gender, age ethnicity, religion, preferred spoken language, highest educational attainment, health conditions, place of residence (e.g., first half of postcode), to help us monitor the range of people we include in this project. Individuals at Newcastle University may look at your research data to check the accuracy of the research study.  The only individuals at Newcastle University who will have access to information that identifies you will be individuals who need to contact you to organise an interview or focus group, update you on any changes to the time or date of your interview or focus group, or audit (review) the data collection process.

If you agree to take part in the research study, information provided by you (such as anonymised quotes) may be shared with researchers running other research studies at Newcastle University and in other organisations.  These organisations may be universities or funding bodies e.g., National Institute for Health and Care Research. Your information will only be used by organisations and researchers to conduct research.

This information will not identify you and will not be combined with other information in a way that could identify you. It will not be used to make decisions about future services available to you.

**What if something goes wrong, who can I contact?**

If you have any questions, would like to receive this information in a different format, font size or language, or would like to reschedule please contact:

- Clare Tolley

Email: [Clare.Brown@newcastle.ac.uk](mailto:Clare.Brown@newcastle.ac.uk)

Phone : 07771397273

To report any formal complaints relating to this study, contact:

- Sarah Slight

Email: [Sarah.Slight@newcastle.ac.uk](mailto:Sarah.Slight@newcastle.ac.uk)

Phone : +44 (0)191 208 6000

This study was approved by the Faculty of Medical Sciences Research Ethics Committee, part of Newcastle University’s Research Ethics Committee. This Committee includes members who are internal to the Faculty. This study was reviewed by members of the Committee, who must provide impartial advice and avoid significant conflicts of interests.
