# Supplementary figures and images for: What are the optimal strategies to communicate the risk of poor air quality exposure to vulnerable groups?
Source: Front Public Health. 2026 Jun 18;14:1763393. doi: 10.3389/fpubh.2026.1763393 (PMC13326810; doi:10.3389/fpubh.2026.1763393)

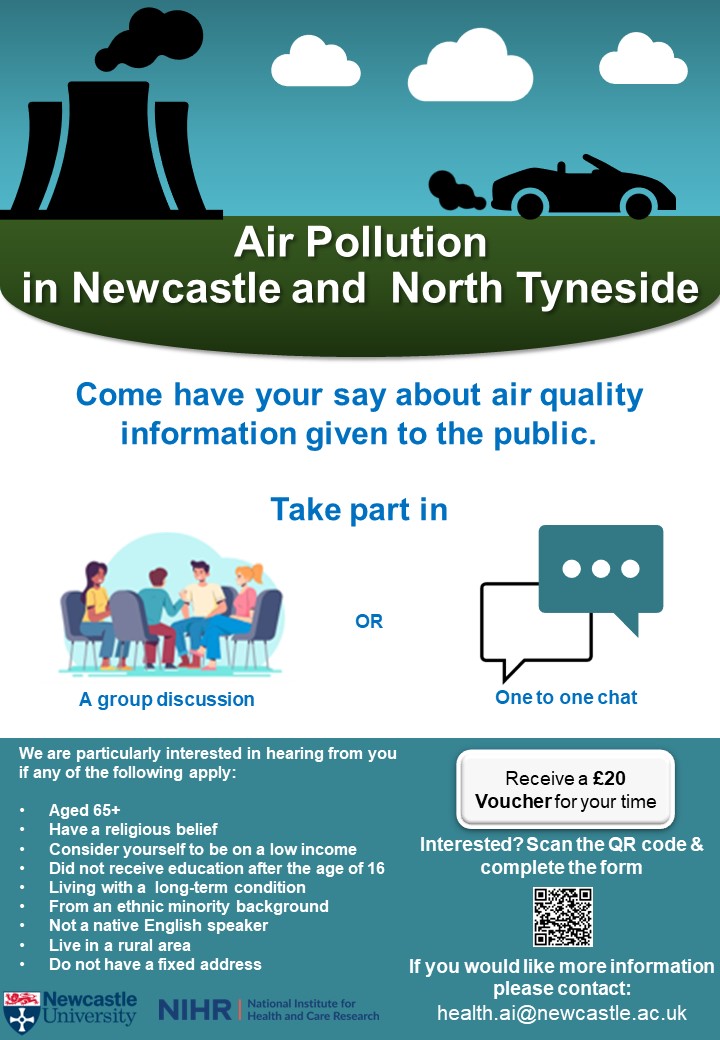

Supplement: Supplementary file 4 [file Image_1.jpeg]

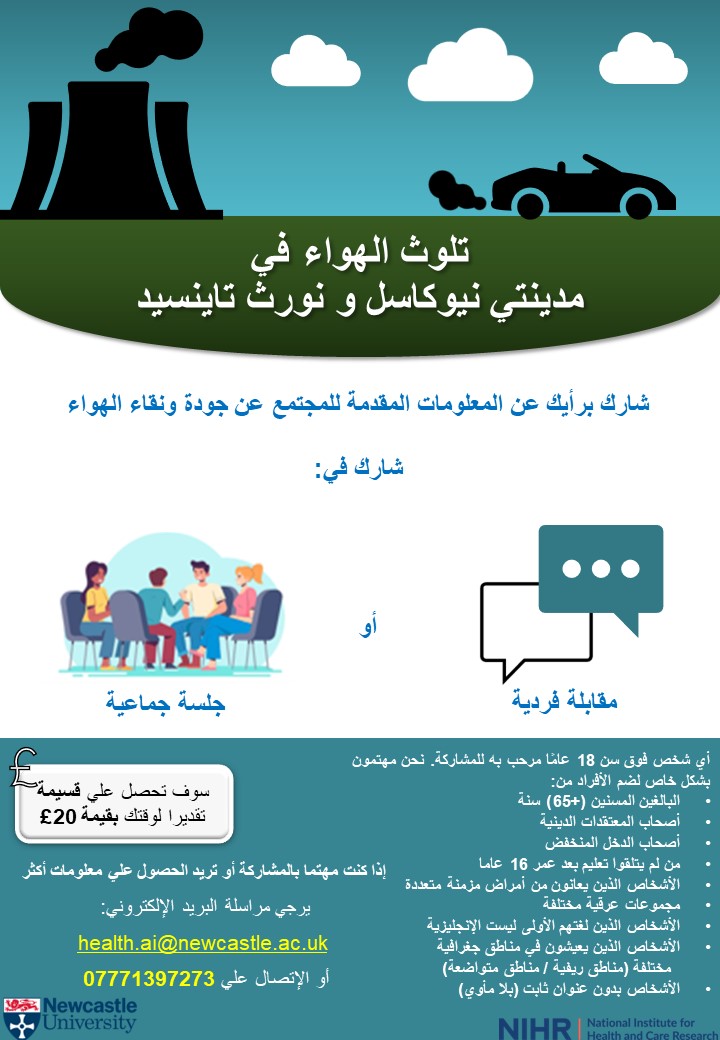

Supplement: Supplementary file 5 [file Image_2.jpeg]
